# Longitudinal plication - a surgical strategy for complete rectal prolapse management

**DOI:** 10.1186/1471-2482-14-17

**Published:** 2014-03-24

**Authors:** Seerwan HS Qaradaghy, Taher AH Hawramy, Beston F Nore, Karwan H-A Abdullah, Rooshad A Muhammad, Mustafa OM Zangana, Jabar M Saleh, Diyaree N Ismael

**Affiliations:** 1 Department of General Surgery, School of Medicine, Faculty of Medical Sciences, University of Sulaimani, Sulaimani, Kurdistan Region, Iraq; 2 Department of General Surgery, Sulaimani Teaching Hospital, Sulaimani, Kurdistan Region, Iraq; 3 Department of Biochemistry, School of Medicine, Faculty of Medical Sciences, University of Sulaimani, Sulaimani, Kurdistan Region, Iraq; 4 Department of Health, Kurdistan Institution for Strategic Studies and Scientific Research, Sulaimaniyah, Kurdistan Region, Iraq; 5 Newham University Hospital, London E13 7SR, UK; 6 Raparin University, Ranya, Kurdistan Region Government, Iraq

**Keywords:** Fecal incontinence, Procidentia, Circumferential protrusion, Rectal wall, Anal sphincter complex

## Abstract

**Background:**

Rectal prolapse is a known problem since antiquity and the cause is not fully understood. Despite the presence of more than 100 lines of treatment, none of them is ideal.

**Methods:**

Between the years of (2005–2011), thirty patients with full-thickness rectal prolapse were operated upon. Age ranged between (2–65 years) with a mean of 21.5 year. Male to female ratio was (2:1). Each prolapsed rectum was repaired with longitudinal plication (LP) at two or three points accordingly using braded polyglycolic acid – absorbable 1.0 suture material. Plications started by inserting a stitch at the most proximal part of the prolapse, followed by successive similar transverse stiches continuing in a spiral fashion till the mucocutaneous junction. We used three LP in adults and two in children. All of the patients where operated upon as a day-case procedure and discharged 6 hours after the operation.

**Results:**

In this series of patients, twenty-nine of them had complete recovery from the prolapse. Only one patient had recurrence 2 years after the operation, and the same procedure was applied successfully with uneventful post-operative period. Although twenty-three patients had fecal Incontinence, twenty-one of them regained continence after operation.

**Conclusions:**

This method is an easy perineal procedure, with fewer complications. It can be performed for all age groups, in an ordinary surgical unit, by an expert anorectal surgeon. We found that our procedure is simple, safe and less invasive.

## Background

The complete rectal prolapse (RP) or procidentia is sliding down of the full thickness of the upper part of the rectum through the anus [1]. It has been known since the Egyptian and Greek times [2]. It is proposed that RP starts as an intussusception of the rectal wall [3] and occurs at the extremes of age [2]. The etiology of RP has not been fully understood till now. Many factors are thought to be the cause, such as increase intra-abdominal pressure, weak anal sphincter, and malnutrition. Other causes include polyps, rectal inflammation, and chronic constipation. In children, straight sacrum and decrease in the angulation between rectum and anus is regarded to be the cause [1]. Rectal prolapse frequently associates with pelvic floor disorders, rectocele and/or enterocele; a condition known as “pelvic floor dysfunction” [2,4]. If it’s not evident; it can be provoked by straining. Rectal prolapse is associated with physical and psychological impacts on the affected patient. The prolapsed part secretes mucus and may results in mucosal ulceration and polyps. It may be associated with incontinence, constipation, or incomplete evacuation with defecation [1].

The Rectal prolapse is diagnosed entirely clinically and the treatment is primarily surgical [5]. The aim is to repair the prolapse with improvement of any associated bowel disorder. More than 100 different techniques have been tried till now. They are either perineal or abdominal approaches. All have a common aim, which is mobilization and fixation of the affected rectum [6]. None of these operations could be regarded ideal for all the patients [7]. Although abdominal approaches have less recurrence rate but significantly associate with higher rate of infection and complications, compared to transperineal approaches [8]. This makes abdominal approaches preferable in medically fit patients. The transperineal approaches is usually reserved for those patients who cannot tolerate the former procedure [9].

Our objective in this paper is to present a novel procedure, which is a perineal approach in treating full-thickness rectal prolapse for all the age-groups. It can be performed as a day-case procedure, regardless of the presence or absence of co morbidities, with fewer complications.

## Methods

### Patient recruitment

At Sulaimani Teaching Hospital (STH), we received 30 patients between the years 2005 and 2011. All had history of full-thickness rectal prolapse for at least 12 months period. The topography of the cases is summarized in Table 1. Male to female ratio was 2:1 and the average age was 21.5 years. Among these 11 were children, 3 adolescences, 15 adults and only one senior, the causes are summarized in Table 2. This research was carried out in accordance with the Declaration of Helsinki (2000) of the World Medical Association. To conduct this study, ethical permission was approved from Ethics Committee at School of Medicine, Faculty of Medical Sciences, University of Sulaimani, Kurdistan Region/Iraq. Full history was taken from each patient or the parents in cases of children. Complete physical examination was performed, including per rectal examination and colonoscopy for all patients to exclude other pathologies. Laboratory tests of hemoglobin level, packed cell volume and viral scan were done. Chest X-ray and electrocardiogram with blood chemistry were done for the adult patients. Written informed consent was taken from each patient, (or the parent in case of children), after a full discussion about this new management, method and the possible sequels. The adult patients were instructed to withhold oral intake, starting midnight before the operation. All of them were advised to evacuate the bowel just before entering the operating theater. The children were kept fasting for 3 hours preoperatively. They were operated upon with a single-handed surgeon, under general anesthesia.

**Table 1:** Age and gender distribution among the patient groups

**Gender**	**Age distribution**			
	**Children (2–10 years)**	**Adolescences (11–17 years)**	**Adults (18–59 years)**	**Seniors (Above 60)**
**Female**	2	3	5	1
**Male**	9	-	10	-
**Total**	11	3	15	1

**Table 2:** The cause in developing rectal prolapse in the patient groups

**Cause**	**Children (2–12)**		**Adolescents (12–17)**		**Adults (18–59)**		**Seniors (≥60)**	
	**Male**	**Female**	**Male**	**Female**	**Male**	**Female**	**Male**	**Female**
Unknown*	5	1	--	3	2	1	--	--
**Constipation**	4	1	--	2	3	1	--	1
**Multi-parity (above 4 pregnancy)**	--	--	--	--	--	3	--	--
**Chronic cough (bronchiectasis due to chemical exposure)**	--	--	--	--	5	1	--	--
**Procidentia**	--	--	--	--	--	--	--	1
**Total**	9	2	--	5	10	6	--	2

*may include oriental toilet style and malnutrition due to multiple embargo over Iraq.

All the operations were carried out as a day-case procedure. The operative time was variable, ranged between 20–61 minutes and the average duration was 31 (±2) minutes. Each patient started oral fluid after four hours from full recovery, and discharged after six hours postoperatively. All the patients were seen again in the follow up clinic one week after the operation, monthly for the next six months and then annually for two years.

### Operative procedure

#### Longitudinal plication (LP)

**Figure 1:**
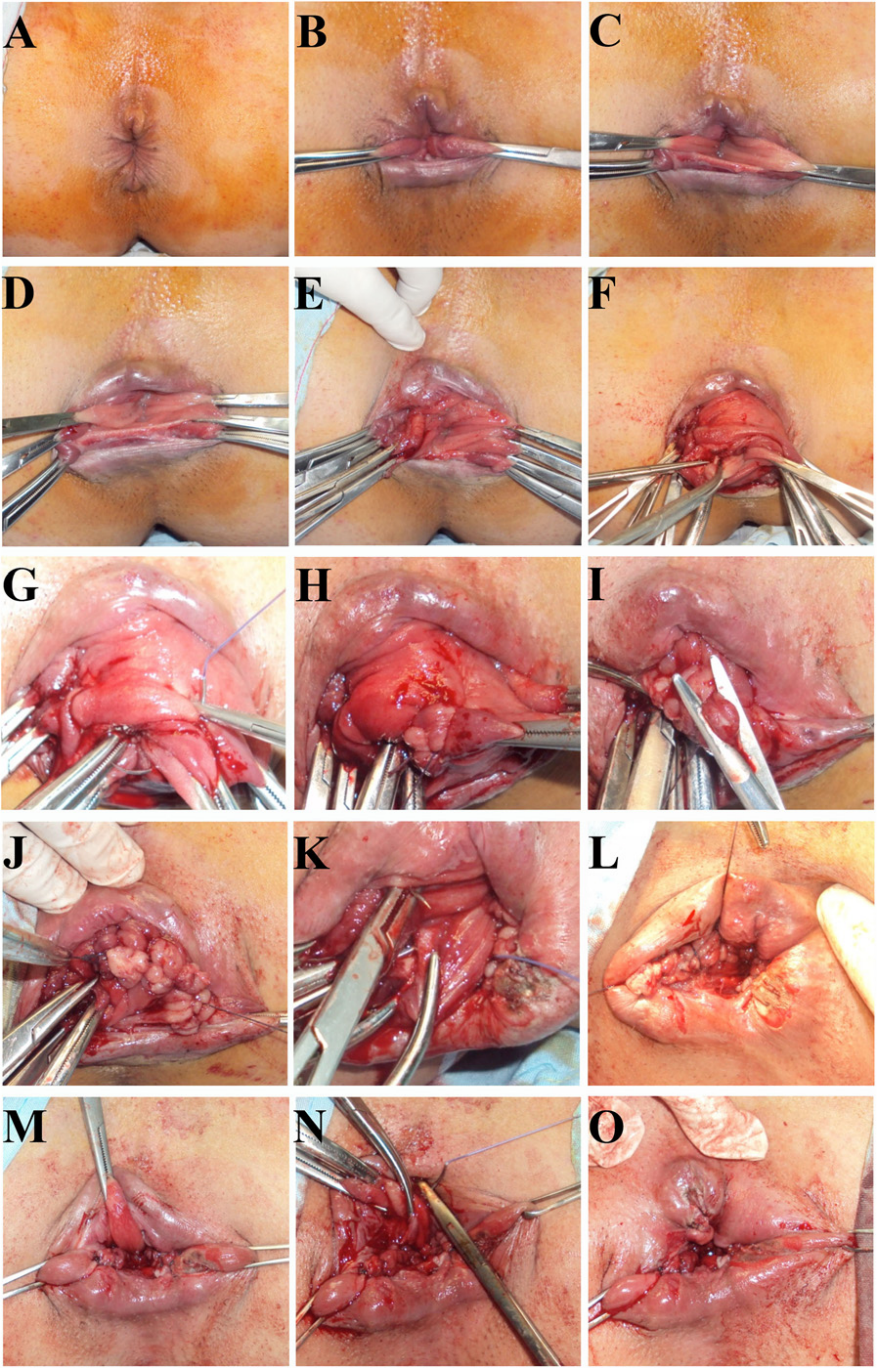
**Longitudinal plication procedure for complete rectal prolapse management. (A)** External view of the anal verge just after induction of the anesthesia before the longitudinal plication. **(B)** The prolapsed area is stretched out by traction and pulling apart through a pair of artery forceps at the mucocutanious junction. **(C-F)** Multiple pairs of artery forceps are used on two-opposite lines in parallel to the long axis of rectum. Step-by-step tractions with these artery forceps makes the prolaps completely exposed. **(G)** The first stitch of the longitudinal plication is inserted just proximal to the tip of the prolapse on the medial aspect at 3:00. **(H)** The longitudinal plication at 3:00 is continued, including 2–3 cm of whole thickness of rectal circumference. **(I)** Residual rectal-wall protrutions between the stitches are excised. **(J)** The longitudinal plication at 3:00 is completed, reaching the mucocutaneous junction. **(K)** The first stitch of the second longitudinal plication is inserted by taking a whole-thickness of the rectal wall medial and proximal to the tip of the prolapsed rectum at 7:00. **(L)** The longitudinal plication at 3:00 and 7:00 are completed. **(M)** The prolapsed part at 11:00 is dragged out. **(N)** The first stitch of the longitudinal plication at 11:00 is inserted, taking a whole-thickness of the rectal wall, at the medial and proximal to the tip of the prolapse. **(O)** The three longitudinal plication pillars at 3:00, 7:00, and 11:00 of the plolapsed rectal wall are completed.

**Figure 2:**
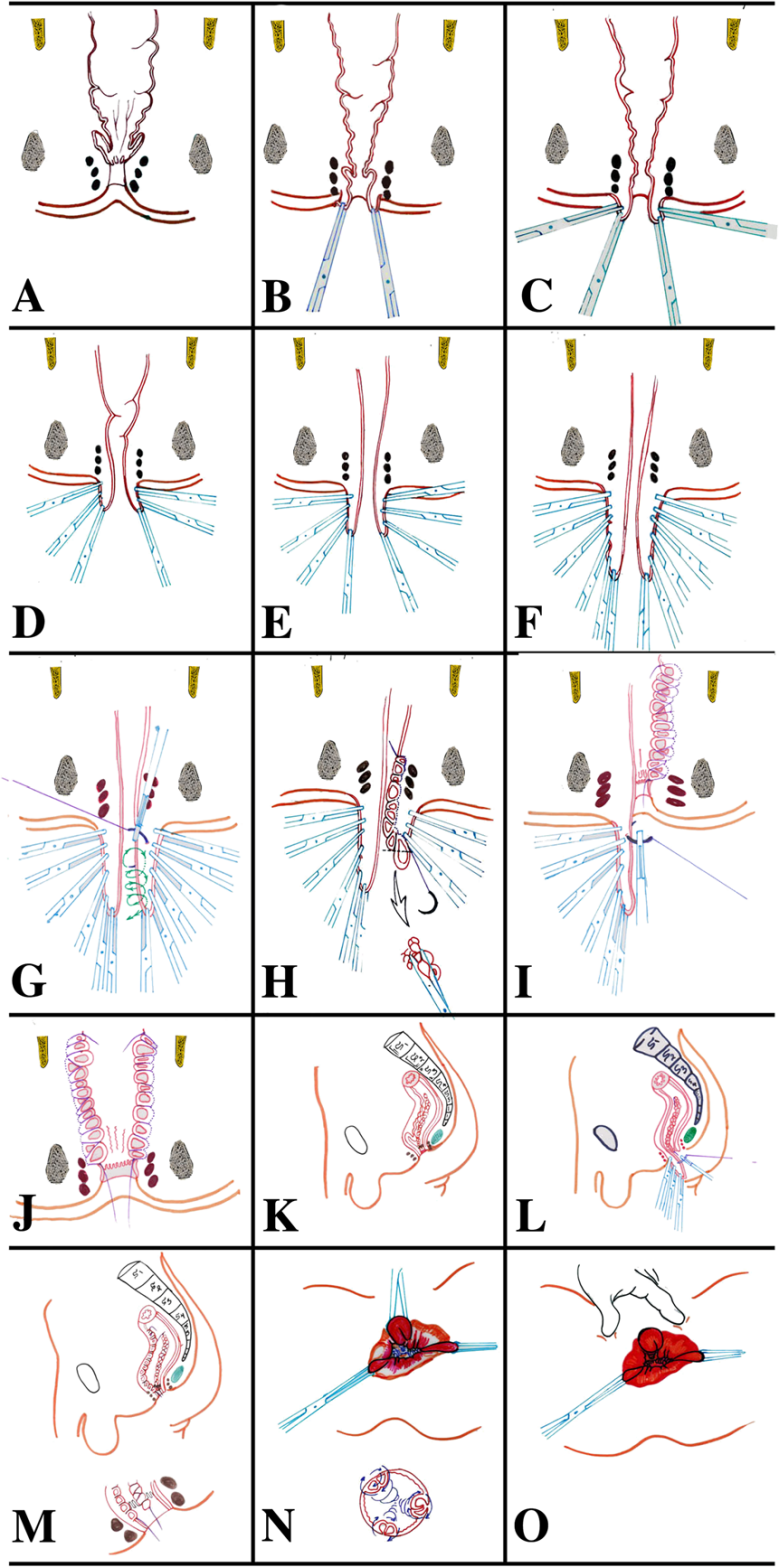
**Diagramatic illustrations of longitudinal plication to explain the sequence of the procedure. (A)** The patient in lithotomy position and the prolapsed rectum is reduced. **(B)** The first step in pulling the prolapse out by traction through a pair of artery forceps fixed at the mucocutaneous junction of the anal canal. **(C-F)** Multiple pairs of artery forceps are used to pull the prolapsed rectum out successively. **(G)** Continous suturing of the first longitudinal plication (first pillar) is started at the most proximal part of the prolapsed rectum involving the entire rectal wall up to the mucocutaneous junction. **(H)** The longitudinal plication at 3:00 is completed and residual rectal-wall protrutions between the stitches are excised. **(I)** The longitudinal plication at 3:00 is completed, creating a pillar and contious suturing for the second pillar at 7:00 is started. **(J)** The second longtitudinal plication at 7:00 (second pillar) is completed. **(K)** sagital section shows the LP on 3:00 is completed and the anterior redundant rectal wall is still in. **(L)** sagital section shows the LP on 3:00 is completed with inserting the first stitch of the L.P. at 11:00, after its traction out through a sets of artery forceps. **(M)** the LPs on 3:00 and 11:00 are completed . **(N)** A cross-section shows a completed pillars at 3.00, 7.00 and 11.00, leaving the normal mucosa between pillars untouched. **(O)** An external view of the anal verge at the end of the procedure at the lithotomy position.

The aim of this surgical technique is to obliterate the redundant rectal wall with subsequent shortening of the wall itself. It also aims to create three longitudinal pillars amend the rectal wall to prevent further intussusception. Prior to the procedure one dose of prophylactic broad spectrum antibiotic was given at induction of the general anesthesia. The patient was put in Lithotomy position (Figure 1A and the corresponding illustration diagram in Figure 2A). In the following steps, Figure 1 shows actual operation images and for clearer illustrations, corresponding diagrams were drawn in Figure 2. Two artery forceps were applied to 3 and 7 O′clock at the mucocutaneous junction (Figures 1B and 2B). This helps as the first step, allowing exteriorization of the prolapsed rectum by mechanical traction. Successive application of artery forceps in a longitudinal line facilitates the process. This mechanical traction and the gravity of the artery forceps help in taking the entire prolapse out (Figures 1C-F and 2C-F).

The second part of the technique is to obliterate the redundant tissue. It comprised inserting a stitch of braded polyglycolic acid – absorbable 1.0 at 3 00 o′clock at the most proximal part of the prolapse. The stitch involves the whole thickness of rectal wall of about 2-3 cm (even 4 cm) of the transverse circumference (Figures 1G and 2G). This is followed by successive 1–1.5 cm apart with similar transverse stitches in a spiral fashion towards the anal verge, ending at the mucocutaneous junction. The artery forceps were successively released and their grips were included in the plication (Figures 1H and 2H). Any protruded rectal mucosa between the stitches was cut to avoid swelling and edema in the lumen (Figures 1I and 2H). The same steps were repeated on 7 and 11 o’clock, reducing the rectal caliber to a point, allowing the surgeon to admit the index and middle finger in adults. After tightening up the rectal wall by these three longitudinal pleats, the wall-circumference shrinks longitudinally and circumferentially. The prolapsed rectal wall relocates (reverse) to its ordinary position, leaving normal wall with its mucosa in between the three tightening pillars (Figures 1J-O and 2I-O). In children two longitudinal plications were quite sufficient, instead of three plications, reducing the rectal caliber to a point admitting the index finger only.

## Results

Thirty patients underwent surgery with no mortality; twenty nine of them reported improvement from their major problem, which was the prolapse. Only one patient, a forty-seven-year-old male, had recurrence after two years from the operation (Clavien- Dindo Grade IIIb). Follow up examination of them showed that there was still redundant rectal wall proximal to our longitudinal plication, which was not included in the first operation. A second surgical operation was carried out using the same procedure, with inclusion of as much as possible from the redundant wall, by three lines of longitudinal plication proximal to the three previous points. Now, he has been free from recurrence for more than two years, since the second operation. Twenty-three patients were suffering from incontinence preoperatively. Twenty-one regained continence, while the remaining two patients reported satisfactory improvement, compared to their condition pre-operatively.

## Discussion

Our aim in this study was to use a less invasive perineal procedure. It can be performed in any surgical center, in all age groups as a day-case surgery. There was no mortality in our patient group in this study (Table 3). We excluded the Thiersch procedure, since it does not treat the real problem and nowadays, has been abandoned [6]. Our method causes minimum inconvenience for the patients and hospital.

In this group, the recurrence rate was 3.33%, comparable and favorable to the abdominal as well as perineal operations (Table 3). The plications shrink the redundant rectal wall longitudinally and circumferentially, stabilizing the rectum. At the same time we buttressed the remaining rectal wall by two or three pillars of plicated full-thickness rectal wall. This prevents future intussusception of any redundant rectal wall left behind, which may be the precursor of future recurrent prolapse on straining [4,9].

There are numerous perineal procedures to treat rectal prolapse. They can broadly be classified into two groups. The first group aims to strengthen the rectal wall through inducing fibrosis, with or without reconstruction of the pelvic floor. They include mucosal cauterization, ligation and excision of the rectal mucosa at different points, or submucosal injection of different materials [1]. In the perineal approach, pioneered by El-Sibai and Shafik on 28 patients [10], they cauterize the rectal mucosa and apply multiple vertical purse-string sutures. Their recurrence rate was 3.57% (n = 1/28). Their idea is to induce fibrosis as well as reducing the prolapsed rectal wall to inside the rectum. They reduce the prolapsed rectum longitudinally only, leaving the redundancy of the rectal wall protruding to inside its cavity. Also the entire pleated rectal wall accumulates near the anal verge. Our method differs through reduction of the redundant rectal wall longitudinally as well as circumferentially. We include the redundant rectal wall in longitudinal plications, keeping an empty space inside the rectum. The pleated rectal wall distributes up through the two or three lines of the longitudinal plications. All the pleated tissue layers are included in the plications, allowing introduction of two figures in adult and one finger in children into the rectal cavity. Our recurrence rate was 3.33% (n = 1/30), which was similar to the results achieved by El-Sibai and Shafik [10].

The second group of the perineal approaches, which have some similarity to our procedure aims to shorten the prolapsed rectum. They include transverse suturing of a longitudinal rectal wall incision, mucosal resection, Delorme’s operation, and perineal amputation of the prolapsed rectum with end-to-end anastomosis. This group also includes stapler rectopexy. Pelvic floor reconstruction may be added to any one of these procedures [1]. The most common performed perineal procedures nowadays are Delorme’s operation, perineal rectosigmoidectomy, and stapled transanal rectal resection [2]. Delorme’s operation plicates the rectal wall muscles submucosally above the anal verge [11]. It requires dissection and has a high recurrence rate as summarized in Table 3[2,6,9].

**Table 3:** Comparison between different plication procedures as percentage of mortality and recurrence

**Complications**	**Mortality %**	**Recurrence %**
**LP**	0	3.33
**Suture rectopexy**	0	0-2.7
**Posterior mesh rectopexy**	0-3	3
**Repstein rectopexy**	0-2.8	0-13
**Resection rectopexy**	0-6.7	0-3
**Anterior resection**	4	4
**Delorme’s plication**	0-4	4-38
**Rectosigmoidectomy (Altimer)**	0-5	0-16

Removing the mass of the redundant and prolapsed rectal wall on the anal ring allows it to regain its turgor and continence [12]. As mentioned above, our procedure does not accumulate the pleated rectal wall on the anal canal, it shifts the redundant wall through the longitudinal plications away from the anus. Each plication ends at the mucocutaneous junction, as we tightened the anal canal and reduced its caliber. We believe this will contribute further to help continence to be regained [9]. Also we have left intact normal mucoca in-between the three longitudinal plications. We can say that our procedure combines both the two previous perineal approaches. We shorten the redundant rectal wall both longitudinally, as well as circumferentially. In addition we also keep the intact rectal mucosa in between the three pillars. This preserves the normal rectal sensation, which is another factor in regaining continence.

Compared to other methods, our technique is a simple perineal procedure, done as a day case surgery. We used this method for all the age groups (Table 1). Traditionally the perineal approaches are reserved for medically unfit patients. They have a higher recurrence rate, compared to the abdominal approaches. Also our procedure is performed through the rectum from inside, involving the rectal wall in the stiches. We didn’t dissect the mucosa, which is associated with excessive bleeding, as well as it is time-consuming. In addition we didn’t disturb the perirectal tissue, which can severe the rectal ligaments; leading to a decrease in the resting and squeezing pressure of the rectum. This may aggravate the pre-existing constipation. Our procedure can be performed, using simple instruments, which are used in any surgical unit [2,3,9].

## Conclusion

The choice of an ideal operation for complete rectal prolapse remains a perplexing problem for surgeons. We prefer longitudinal plication of the whole rectal wall through a perineal approach for all ages. Although the number of patients was limited in this study, we noticed a far-more beneficial recovery from rectal prolapse problems for all patient groups and a better functional outcome toward incontinence symptoms as well.

### Consent

Patients and their families were informed and written consents were obtained and the anonymity of patient’s identities preserved. Copies of the consent documents are available for review by the Editor-in-Chief of this journal.

## Competing interest

The authors declare that they have no competing interests.

## Authors’ contributions

SHSQ and TAHH designed the project and coordinated the overall project. They performed all clinical work, surgery, treatment, and follow-up of the patient with the families. DNI wrote the manuscript. BFN contributed in writing, editing and restructuring the style of the manuscript. KH-AA, RAM and JMS contributed in data preparations, including illustrations and tables. MOMZ was consulted on alternative clinical procedures and contributed in writing and editing. All authors read and approve the manuscript.

## Pre-publication history

The pre-publication history for this paper can be accessed here:

http://www.biomedcentral.com/1471-2482/14/17/prepub
